# Sex-specific trends in incidence of first myocardial infarction among people with and without diabetes between 1985 and 2016 in a German region

**DOI:** 10.1186/s12933-024-02179-1

**Published:** 2024-03-30

**Authors:** Heiner Claessen, Maria Narres, Margit Heier, Tatjana Kvitkina, Birgit Linkohr, Georg Wolff, Michael Roden, Andrea Icks, Annette Peters

**Affiliations:** 1 Institute for Health Services Research and Health Economics, German Diabetes Center (DDZ), Leibniz Center for Diabetes Research at Heinrich-Heine-University Düsseldorf, Auf´m Hennekamp 65, 40225 Düsseldorf, Germany; 2 Institute for Health Services Research and Health Economics, Center for Health and Society, Faculty of Medicine, Heinrich Heine University, Düsseldorf, Germany; 3 German Center for Diabetes Research (DZD), Munich-Neuherberg, Germany; 4 Institute of Epidemiology, Helmholtz Zentrum München – German Research Center for Environmental Health (GmbH), Neuherberg, Germany; 5 KORA Study Centre, University Hospital, Augsburg, Germany; 6 Clinic of Cardiology, Pulmonology, and Vascular Medicine, Medical Faculty of the Heinrich, Heine University, Düsseldorf, Germany; 7 Division of Endocrinology and Diabetology, Medical Faculty, Heinrich Heine University, University Hospital, Düsseldorf, Germany; 8 Institute for Clinical Diabetology, Leibniz Center for Diabetes Research at Heinrich Heine University Düsseldorf, German Diabetes Center, Düsseldorf, Germany; 9 Chair of Epidemiology, Institute for Medical Information Processing, Biometry and Epidemiology, Medical Faculty, Ludwig-Maximilians-Universität München, Munich, Germany; 10 DZHK (German Center for Cardiovascular Research), partner site Munich Heart Alliance, Munich, Germany

**Keywords:** Myocardial infarction, Diabetes mellitus, Incidence, Sex difference, Population-based study, Time trend

## Abstract

**Background:**

The reduction of myocardial infarction (MI) and narrowing the gap between the populations with and without diabetes are important goals of diabetes care. We analyzed time trends for sex-specific incidence rates (IR) of first MI (both non-fatal MI and fatal MI) as well as separately for first non-fatal MI and fatal MI in the population with and without diabetes.

**Methods:**

Using data from the KORA myocardial infarction registry (Augsburg, Germany), we estimated age-adjusted IR in people with and without diabetes, corresponding relative risks (RR), and time trends from 1985 to 2016 using Poisson regression.

**Results:**

There were 19,683 people with first MI (34% fatal MI, 71% men, 30% with diabetes) between 1985 and 2016. In the entire study population, the IR of first MI decreased from 359 (95% CI: 345–374) to 236 (226–245) per 100,000 person years. In men with diabetes, IR decreased only in 2013–2016. This was due to first non-fatal MI, where IR in men with diabetes increased until 2009–2012, and slightly decreased in 2013–2016. Overall, fatal MI declined stronger than first non-fatal MI corresponding to IRs. The RR of first MI substantially increased among men from 1.40 (1.22–1.61) in 1985–1988 to 2.60 (2.26–2.99) in 1997–2000 and moderately decreased in 2013–2016: RR: 1.75 (1.47–2.09). Among women no consistent time trend for RR was observed. Time trends for RR were similar regarding first non-fatal MI and fatal MI.

**Conclusions:**

Over the study period, we found a decreased incidence of first MI and fatal MI in the entire study population. The initial increase of first non-fatal MI in men with diabetes needs further research. The gap between populations with and without diabetes remained.

## Background

The 1989 St. Vincent Declaration defined several major targets for improving diabetes care. One primary objective was narrowing the gap in cardiovascular morbidity between the populations with and without diabetes [1]. Several studies have shown a declining incidence rate (IR) of cardiovascular events, e.g. myocardial infarction (MI) in people with diabetes since the 1990s [2]. However, only some studies compared trends for cardiovascular event rates in populations with and without diabetes and analysed secular trends for the corresponding relative risk (RR) [2]. Hence, it is still unclear whether there has been a proportionate decline in cardiovascular events in people with and without diabetes or whether the gap between the populations with and without diabetes has been narrowed. The majority of studies comparing MI among people with and without diabetes only reported incidence of non-fatal MI based on hospital admission data [3–6]. Moreover, the few available studies that analysed both non-fatal MI and fatal events did not stratify results by MI fatality and showed contradictory time trend results with some sex-specific differences. One Finnish study comparing IR of MI in two cohorts with a 10-year follow-up found a decreased time trend in men with diabetes and in men and women without diabetes, but no change among women with diabetes [7]. A U.S. study found corresponding results [8]. Conversely, a Swedish study did not identify any consistent time trends in men and women with diabetes or in women without diabetes between 1989 and 2000, but a significant decrease in men without diabetes [9]. A recent Scottish study covering 2006–2015 reported a significant decrease of IR of MI with approx. 2% reduction per year both in people with and without diabetes with no sex differences [10]. In our previous study IR of MI decreased between 1985 and 2006 in men and women without diabetes and in women with diabetes, whilst it increased in men with diabetes [11]. Due to inconsistent results concerning secular trends for MI, it remained of great interest to repeat this analysis to evaluate more recent time trends using the same MI data sources. The objectives of this study were therefore to (i) estimate a sex-specific IR of first MI (both non-fatal MI and fatal MI) as well as separately for first non-fatal MI and fatal MI in the population with and without diabetes, and RRs comparing these populations’ IR and (ii) analyse their time trends between 1985 and 2016.

## Methods

### Study design and study population

We used data from the KORA (Cooperative health research in the region of Augsburg) myocardial infarction registry from the region of Augsburg, Germany (approx. 600,000 inhabitants from the city of Augsburg and the two adjacent districts Augsburg and Aichach-Friedberg) to identify people with first MI. Case finding, diagnostic classification of events and data quality control methods are described elsewhere [12–14]. The population-based Augsburg MI registry was implemented in 1984 as part of the WHO MONICA project [12, 13, 15]. It was part of the KORA platform from 1996 until 2019 and continues as Augsburg Myocardial Infarction Register from 2020. It continuously registered all cases of first MI (non-fatal MI and fatal MI) in the study population [13].

Data from four population-based surveys and two follow-up surveys conducted in the study region of the KORA MI registry are available. The first three surveys were part of the MONICA project (S1:1984/1985, S2:1989/1990, S3:1994/1995). The fourth (S4:1999–2001) and the follow-up surveys of S3 (F3: 2004/2005) and S4 (F4: 2006–2008) were conducted as KORA studies. These surveys were used to define the background population with and without diabetes. The baseline survey population of S1 was between 25 and 64 years old and between 25 and 74 years old in S2, S3 and S4. The survey was carried out using cluster sampling procedures with a response rate between 79% in S1 [16] and 68% in S4 [17]. In the F3 survey (survey population 35–84 years old) 76% of all eligible S3-participants responded [18] while in F4 (survey population 32–81 years old) 80% of all eligible S4 participants made their response [19].

Moreover, data from the Central Research Institute of Ambulatory Health Care (Zi-data) in Germany, available since 2009, were used to estimate the population with diabetes between 2009 and 2016 [20]. The Zi-data comprises all people with statutory health insurance having at least one medical contact in a calendar year. Over 80% of the total study region population were covered.

The present study excluded all people aged between 25 and 44 since no valid diabetes prevalence estimates were available for that age group for the study period. Thus, the remaining study population (45 to 74 years) ranged from 179,689 people in 1985 to 254,773 in 2016.

### Definition of MI cases and data assessment

MI definition methods are described in detail elsewhere [11, 21]. Briefly, all cases of non-fatal MI and fatal MI between January 1, 1985 and December 31, 2016 were recorded according to the WHO MONICA protocol [12, 14].

For this study the clinical diagnosis of non-fatal MI was categorized and validated according to MONICA criteria [12], which included acute clinical symptoms (acute chest pain lasting 20 min or longer, not relieved by rest or nitrates), ECG diagnostic criteria (Q waves, non-Q waves in up to four electrocardiograms) as well as a subsequent increase in the serum activity of at least one of three enzymes (creatinine phosphokinase, aspartate aminotransferase, and lactate dehydrogenase) of more than twice the normal upper limit. Since January 1, 2001, non-fatal MI has been diagnosed according to the European Society of Cardiology and American College of Cardiology criteria [22]. An MI case was defined as non-fatal if a patient reached the hospital and survived for at least 24 h. Patients were additionally interviewed during hospitalization using a standardized questionnaire including factors such as previous myocardial infarction. Further data were gathered in a concluding chart review. We only analysed first cases of non-fatal MI in the current study.

Fatal MI was defined as cases of coronary death or early fatal MI occurring before or within 24 h of hospitalization if no previous non-fatal MI was recorded. Coronary deaths were identified via regional health offices by checking all death certificate diagnoses giving suspected coronary heart disease as main cause of death. Additionally, a written questionnaire enquiring about disease history (in particular previous myocardial infarction), prior medication, risk factors, and circumstances of death was routinely sent to the deceased person’s last treating physician and the coroner (mean response 85%).

First MI was defined as the sum of first non-fatal MI and fatal MI.

All patients with first MI were assessed by age, sex, date of first MI, and diabetes status.

### Definition of diabetes

Diabetes in people with first non-fatal MI was defined by a physician’s diagnosis assessed from chart review (99.7% of the cases) or by self-reporting in a personal interview (0.3%). Diabetes status in case of fatal MI was obtained from questionnaires sent to the last treating physicians or coroner. All people with unclear diabetes status were excluded from the analysis (*n* = 300).

Diabetes among the population at risk: KORA surveys classified a person as having diabetes based on self-reported physician’s diabetes diagnosis or reported use of glucose-lowering medication. The Zi-dataset classified someone as having diabetes if an outpatient diagnosis was made in at least two quarters of a calendar year.

### Statistical analysis

All main analyses were performed for the entire population, stratified by sex, and for first MI as well as separately for, first non-fatal MI and fatal MI. For diabetes population definition, we estimated the age- and sex-specific prevalence of diabetes for each calendar year using the age classes 45–54, 55–64 and 65–74 years based on combined KORA surveys and Zi-data. Age-sex specific diabetes prevalence in the KORA survey in 2013/2014 was as follows: men: 2.0% (45–54 years), 8.7% (55–64 years), 19.8% (65–74 years); women: 2.9% (45–54 years), 6.4% (55–64 years), 11.9% (65–74 years). The mean of the age-sex specific diabetes prevalence in the Zi-data in 2013 and 2014 was as follows: men: 7.1% (45–54 years), 16.5% (55–64 years), 27.4% (65–74 years); women: 4.1% (45–54 years), 11.0% (55–64 years), 19.8% (65–74 years).

A correction factor was calculated for each age and sex stratum by dividing the mean prevalence from the Zi-data (years 2013 and 2014) by the 2013/2014 KORA prevalence. We then multiplied all age- and sex-specific KORA prevalence estimates by this correction factor. The corrected KORA prevalence estimates for the years of the surveys (1984/85, 1989/90, 1994/95, 1999–2001, 2004/2005, 2006–2008) were used to calculate diabetes prevalence between 1985 and 2008, assuming a linear time trend between the survey years. Age- and sex-specific diabetes prevalence was estimated directly from the Zi-data for the time period 2009 to 2016.

The population with diabetes was estimated for each stratum (age class, sex, and calendar year of first MI) by multiplying the population of the study areas by the age- and sex-specific prevalence of diabetes as described above. The population without diabetes was estimated by subtracting this population from the total population in each stratum.

In the sensitivity analysis using only the KORA prevalence, a linear trend for the age- and sex-specific diabetes prevalence was assumed between the surveys. Moreover, it was assumed that this prevalence remained constant in the years after the last survey (i.e., 2015 and 2016).

Calendar time periods were aggregated as follows to account for random fluctuations in the individual years of first MI: 1985–1988, 1989–1992, 1993–1996, 1997–2000, 2001–2004, 2005–2008, 2009–2012 and 2013–2016.

Stratum-specific and age- and sex-standardized MI IRs were calculated for each time period and for both populations with and without diabetes. We used the German population from the year 2000 as standard population.

Incidence rate ratios (population with vs. without diabetes) were estimated from the standardized IRs.

To test for time trends, we performed Poisson regression models using calendar period of MI IR, age (classes as described above) and sex as independent variables. We used the calendar period 1985–1988 as a reference to estimate the effect of calendar time. Furthermore, analogous Poisson models were fitted including a variable presence of diabetes (yes vs. no) stratified by calendar period of first MI. The change of the RR between time periods using 1985–1988 as baseline period was statistically tested including a variable presence of diabetes (yes vs. no) and an interaction term “diabetes*calendar period of first MI”.

The main analyses were repeated in a sensitivity analysis, whereby only KORA diabetes prevalence was used (detailed information see appendix). Another sensitivity analysis estimated linear time trend for the years 2009–2016 using only the Zi-data for each calendar year.

All analyses were performed using de-scale adjustment to account for over-dispersion of the dependent variable [23]. We performed all analyses using the statistical analysis systems SAS (SAS for Windows 10, Release 9.4 TS1M5, SAS Institute, Cary, NC, USA).

### Ethics approval

The KORA MI Registry and KORA survey data collection has been approved by the ethics committee of the Bavarian Medical Association (Bayerische Landesärztekammer, Germany). All survey participants and MI patients who actively participated in the registry gave written consent. The study was performed according to the principles of good epidemiological practice [24].

## Results

### Study population

Table 1 presents a description of the study population. The average study population increased from 179,689 in 1985–1988 to 254,773 in 2013–2016. Diabetes prevalence in people aged 45–74 years was 11.6% in the first study period 1985–1988 and 12.9% in the last study period 2013–2016. In total, we identified 19,683 people with a first MI between 1985 and 2016. About one third had a fatal MI, 71.0% were men, and on average 29.9% had diabetes at first diagnosis of MI. The proportion of fatal MI was higher among people with diabetes, but decreased consistently during the study period (1985–1988: diabetes: 49.3%, no diabetes 40.4%; 2013–2016: diabetes: 24.9%, no diabetes: 17.3%).

**Table 1:** Description of all people with first myocardial infarction and the background population, Augsburg. MONICA/KORA region, 1985–2016

Characteristic	Total	Men	Women	Diabetes	No diabetes	Men			Women	
						Diabetes	No diabetes		Diabetes	No diabetes
**All years combined**										
Number of people with first MI^a^	19,683	13,981	5,702	5890 (29.9%)	13,793 (70.1%)	3907 (27.9%)	10,074 (72.1%)		1983 (34.8%)	3719 (65.2%)
Mean age^b^ (years [SD^c^])	63.1 (8.0)	62.1 (8.1)	65.4 (7.4)	64.8 (7.3)	62.3 (8.2)	63.8 (7.5)	61.5 (8.2)		66.8 (6.5)	64.7 (7.7)
45–54	3563	2935	628	680 (19.1%)	2883 (80.9%)	548 (18.7%)	2387 (81.3%)		132 (21.0%)	496 (79.0%)
55–64	6288	4833	1455	1733 (28.2%)	4555 (71.8%)	1287 (26.6%)	3546 (73.4%)		446 (30.7%)	1009 (69.3%)
65–74	9832	6213	3619	3477 (35.4%)	6355 (64.6%)	2072 (33.3%)	4141 (66.7%)		1405 (38.9%)	2214 (61.1%)
Type of first MI										
First non-fatal MI	13,054	9634	3420	3609 (27.6%)	9445 (82.4%)	2515 (26.1%)	7119 (73.9%)		1094 (32.0%)	2326 (68.0%)
Fatal MI	6629	4347	2282	2281 (34.4%)	4348 (65.6%)	1392 (32.0%)	2955 (68.0%)		889 (39.0%)	1393 (61.0%)
**Period 1985–1988**										
Number of people with first MI	2330	1611	719	572 (24.5%)	1758 (75.5%)	306 (19.0%)	1305 (81.0%)		266 (37.0%)	453 (63.0%)
Mean age^b^ (years [SD^c^])	63.1 (8.0)	61.8 (8.2)	66.1 (6.7)	65.7 (6.9)	62.3 (8.2)	63.9 (7.6)	61.3 (8.3)		67.7 (5.3)	65.2 (7.3)
First non-fatal MI	1337	995	342	290 (21.7%)	1047 (78.3%)	169 (17.0%)	826 (83.0%)		121 (35.4%)	221 (64.6%)
Fatal MI	993	616	377	282 (28.4%)	711 (71.6%)	137 (22.2%)	479 (77.8%)		145 (38.5%)	232 (61.5%)
Population at risk^d^	179,689	81,586	98,103	20,878 (11.6%)	158,811 (88.4%)	10,983 (13.5%)	70,604 (86.5%)		9896 (10.1%)	88,207 (89.9%)
**Period 1989–1992**										
Number of people with first MI	2463	1645	818	672 (27.3%)	1791 (72.7%)	390 (23.7%)	1255 (76.3%)		282 (34.5%)	536 (65.5%)
Mean age^b^ (years [SD^c^])	63.2 (7.7)	62.0 (7.8)	65.5 (6.8)	65.0 (7.0)	62.5 (7.8)	63.8 (7.3)	61.5 (7.9)		66.6 (6.1)	65.0 (7.0)
First non-fatal MI	1348	946	402	302 (22.4%)	1046 (77.6%)	188 (19.9%)	758 (80.1%)		114 (28.4%)	288 (71.6%)
Fatal MI	1115	699	416	370 (33.2%)	745 (66.8%)	202 (28.9%)	497 (71.1%)		168 (40.4%)	248 (59.6%)
Population at risk^d^	189,030	88,388	100,642	25,741 (13.6%)	163,289 (86.4%)	14,870 (16.8%)	73,517 (83.2%)		10,871 (10.8%)	89,772 (89.2%)
**Period 1993–1996**										
Number of people with first MI	2393	1615	778	702 (29.3%)	1691 (70.7%)	425 (26.3%)	1190 (73.7%)		277 (35.6%)	501 (64.4%)
Mean age^b^ (years [SD^c^])	63.7 (7.8)	62.6 (7.8)	66.0 (7.2)	65.2 (7.2)	63.1 (7.9)	64.1 (7.3)	62.0 (7.9)		66.9 (6.9)	65.5 (7.4)
First non-fatal MI	1340	953	387	328 (24.5%)	1012 (75.5%)	201 (21.1%)	752 (78.9%)		127 (32.8%)	260 (67.2%)
Fatal MI	1053	663	391	374 (35.5%)	679 (64.5%)	224 (33.8%)	438 (66.2%)		150 (38.4%)	241 (61.6%)
Population at risk^d^	200,818	95,433	105,386	25,351 (12.6%)	175,467 (87.4%)	14,081 (14.8%)	81,352 (85.2%)		11,270 (10.7%)	94,116 (89.3%)
**Period 1997–2000**										
Number of people with first MI	2522	1807	715	777 (30.8%)	1745 (69.2%)	520 (28.8%)	1287 (71.2%)		257 (35.9%)	458 (64.1%)
Mean age^b^ (years [SD^c^])	63.4 (7.9)	62.2 (7.9)	66.3 (6.9)	65.1 (6.9)	62.7 (8.2)	63.8 (7.0)	61.6 (8.2)		67.5 (6.1)	65.7 (7.3)
First non-fatal MI	1426	1093	333	402 (28.2%)	1024 (71.8%)	301 (27.5%)	792 (72.5%)		101 (30.3%)	232 (69.7%)
Fatal MI	1096	714	382	375 (34.2%)	721 (65.8%)	219 (30.7%)	495 (69.3%)		156 (40.8%)	226 (59.2%)
Population at risk^d^	206,488	99,793	106,696	22,322 (10.8%)	184,166 (89.2%)	12,426 (12.5%)	87,367 (87.5%)		9896 (9.3%)	96,800 (90.7%)
**Period 2001–2004**										
Number of people with first MI	2625	1917	708	858 (32.7%)	1767 (67.3%)	599 (31.2%)	1318 (68.8%)		259 (36.6%)	449 (63.4%)
Mean age^b^ (years [SD^c^])	63.3 (7.8)	62.4 (7.8)	65.7 (7.1)	65.1 (7.0)	62.4 (8.0)	64.1 (7.2)	61.6 (8.0)		67.5 (5.9)	64.6 (7.6)
First non-fatal MI	1883	1387	496	579 (30.7%)	1304 (69.3%)	407 (29.3%)	980 (70.7%)		172 (34.7%)	324 (65.3%)
Fatal MI	742	530	212	279 (37.6%)	463 (62.4%)	192 (36.2%)	338 (63.8%)		87 (41.0%)	125 (59.0%)
Population at risk^d^	216,546	105,686	110,860	27,412 (12.7%)	189,134 (87.3%)	15,810 (15.0%)	89,876 (85.0%)		11,602 (10.5%)	99,258 (89.5%)
**Period 2005–2008**										
Number of people with first MI	2486	1809	677	801 (32.2%)	1685 (67.8%)	554 (30.6%)	1255 (69.4%)		247 (36.5%)	430 (63.5%)
Mean age^b^ (years [SD^c^])	63.0 (8.1)	62.3 (8.1)	64.7 (7.8)	64.3 (7.4)	62.4 (8.3)	63.6 (7.4)	61.8 (8.3)		65.9 (7.1)	64.0 (8.1)
First non-fatal MI	1908	1404	504	584 (30.6%)	1324 (69.4%)	401 (28.6%)	1003 (71.4%)		183 (36.3%)	321 (63.7%)
Fatal MI	578	405	173	217 (37.5%)	361 (62.5%)	153 (37.8%)	252 (62.2%)		64 (37.0%)	109 (63.0%)
Population at risk^d^	230,418	113,058	117,361	29,591 (12.8%)	200,827 (87.2%)	16,060 (14.2%)	96,998 (85.8%)		13,532 (11.5%)	103,829 (88.5%)
**Period 2009–2012**										
Number of people with first MI	2526	1843	683	828 (32.8%)	1698 (67.2%)	619 (33.6%)	1224 (66.4%)		209 (30.6%)	474 (69.4%)
Mean age^b^ (years [SD^c^])	62.7 (8.5)	62.1 (8.5)	64.4 (8.2)	64.2 (8.0)	62.0 (8.6)	63.7 (7.9)	61.2 (8.6)		65.6 (8.0)	63.9 (8.3)
First non-fatal MI	1930	1435	495	613 (31.8%)	1317 (68.2%)	468 (32.6%)	967 (67.4%)		145 (29.3%)	350 (70.7%)
Fatal MI	596	408	188	215 (36.1%)	381 (63.9%)	151 (37.0%)	257 (63.0%)		64 (34.0%)	124 (66.0%)
Population at risk^d^	245,225	120,630	124,595	30,967 (12.6%)	214,258 (87.4%)	18,016 (14.9%)	102,615 (85.1%)		12,952 (10.4%)	111,643 (89.6%)
**Period 2013–2016**										
Number of people with first MI	2338	1734	604	680 (29.1%)	1658 (70.9%)	494 (28.5%)	1240 (71.5%)		186 (30.8%)	418 (69.2%)
Mean age^b^ (years [SD^c^])	62.3 (8.3)	61.6 (8.3)	64.3 (8.0)	64.2 (7.6)	61.5 (8.5)	63.6 (7.8)	60.8 (8.4)		66.0 (6.6)	63.5 (8.4)
First non-fatal MI	1882	1421	461	511 (27.2%)	1371 (72.8%)	380 (26.7%)	1.041 (73.3%)		131 (28.4%)	330 (71.6%)
Fatal MI	456	313	143	169 (37.1%)	287 (62.9%)	114 (36.4%)	199 (63.6%)		55 (38.5%)	88 (61.5%)
Population at risk^d^	254,773	125,592	129,180	32,888 (12.9%)	221,884 (87.1%)	19,191 (15.3%)	106,402 (84.7%)		13,698 (10.6%)	115,483 (89.4%)

^a^ Myocardial infarction

^b^ Measured at the time of Myocardial infarction

^c^ Standard deviation

^d^ Data of the population at risk collected from official statistics of the Federal Statistical Office, Population with and without diabetes estimated by means of data from the KORA Surveys (1985–2008) and data from the Central Research Institute of Ambulatory Health Care (Zi-data) (2009–2016)

### Incidence rates and relative risk

**Fig. 1:**
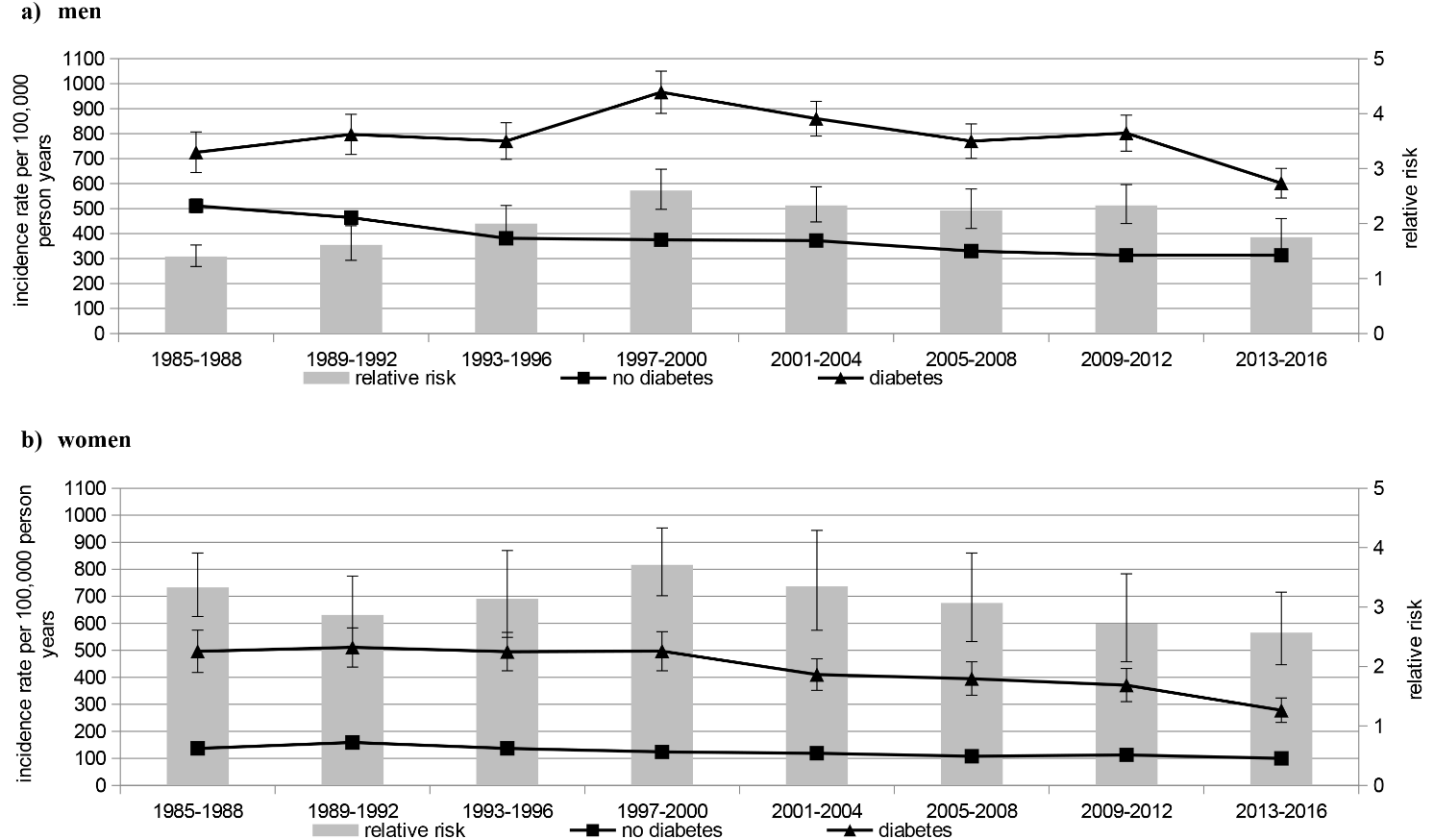
Incidence rate and relative risk of first myocardial infarction (both non-fatal and fatal myocardial infarction)

**Fig. 2:**
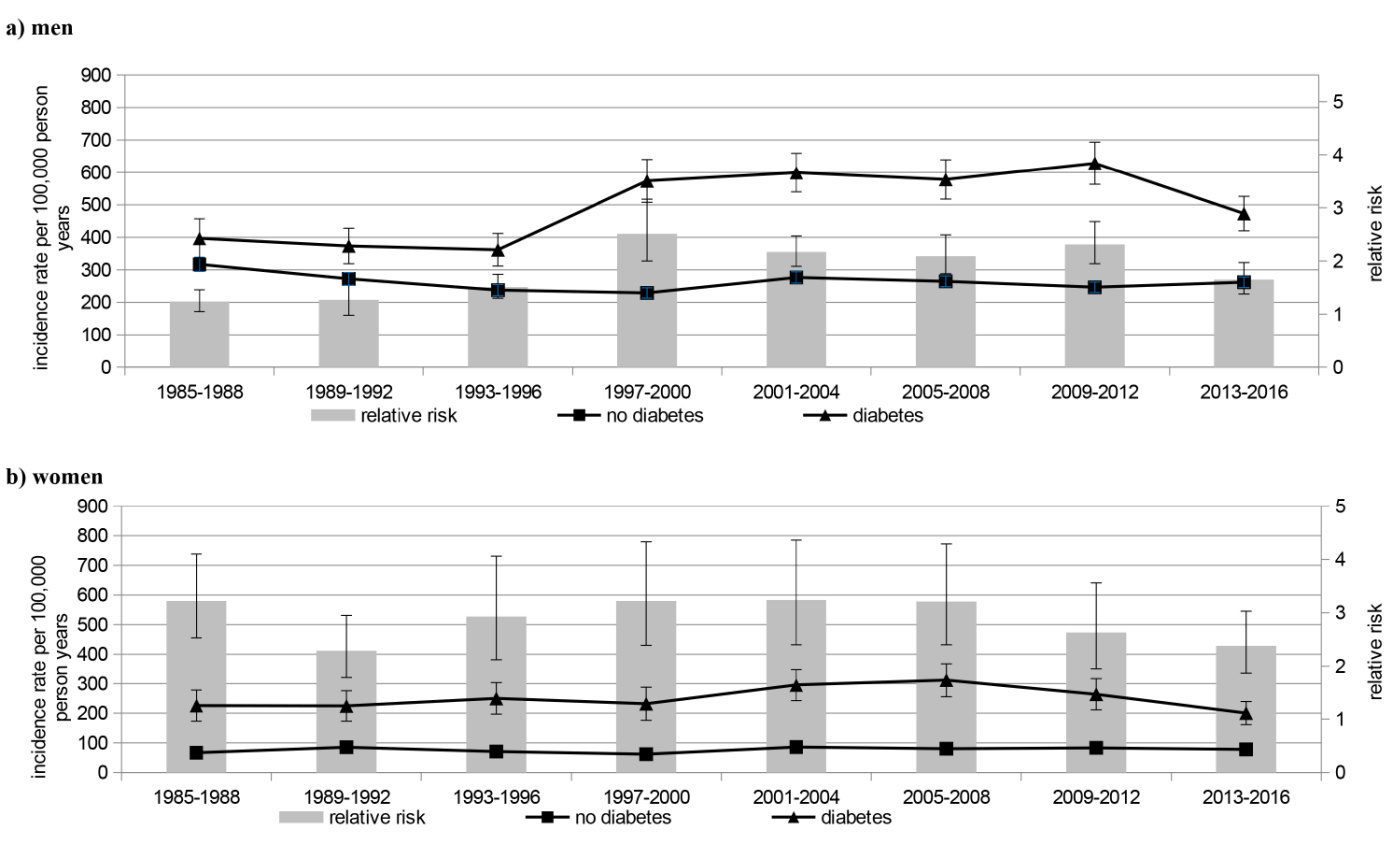
Incidence rate and relative risk of first non-fatal myocardial infarction

**Fig. 3:**
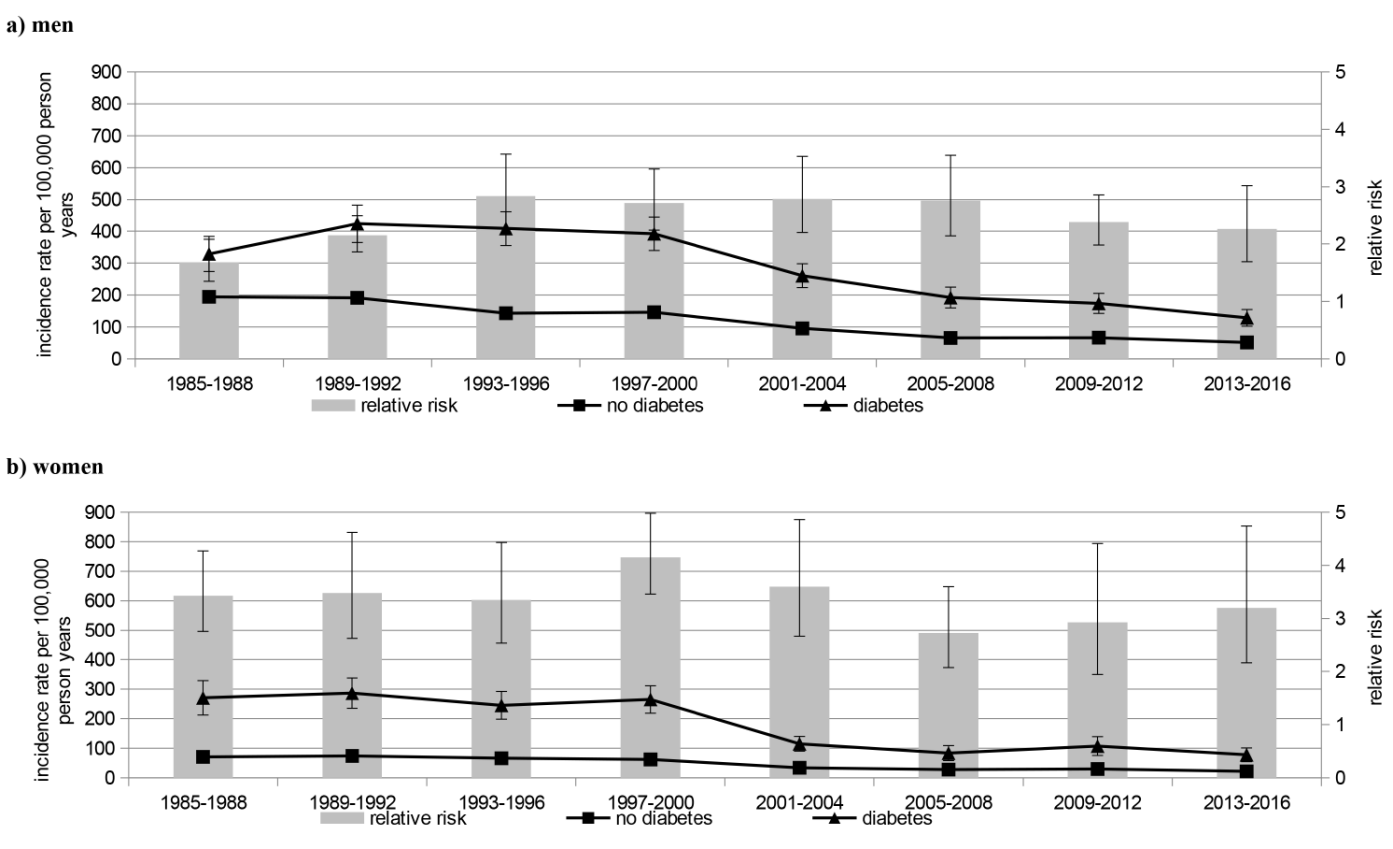
Incidence rate and relative risk of fatal myocardial infarction

Mean IR of first MI in people with diabetes during the study period was 572 per 100,000 person years with a higher rate among men (men: 744, women: 416). In people without diabetes this IR was considerably lower (245 per 100,000 person years) with higher numbers in men (men: 380, women: 120). The RR comparing people with and without diabetes was 2.34 (95% confidence interval 2.26–2.42) and was more pronounced among women than men: incidence rate ratio: men: 1.96 (1.89–2.03), women: 3.47 (3.26–3.70) (see Table 2; Fig. 1 for results). These patterns were similar for first non-fatal MI and fatal MI (Table 2; Figs. 2 and 3).

**Table 2:** Incidence of first myocardial infarction, Augsburg, MONICA/KORA region, 1985–2016

IRs^a^ (95% CI) per 100.000 PY				
	IRt	IRd	IRn	IRR
First MI (both non-fatal MI and fatal MI)				
**All years combined**				
Total	293 (289–297)	572 (556–588)	245 (241–249)	2.34 (2.26–2.42)
Men	441 (434–448)	744 (720–767)	380 (372–387)	1.96 (1.89–2.03)
Women	156 (152–160)	416 (393–439)	120 (116–124)	3.47 (3.26–3.70)
**Men and women - stratified by period**				
1985–1988	359 (345–374)	608 (551–664)	319 (304–334)	1.90 (1.71–2.11)
1989–1992	355 (341–370)	650 (596–704)	307 (293–322)	2.12 (1.92–2.33)
1993–1996	307 (294–319)	629 (578–680)	256 (243–268)	2.46 (2.24–2.70
1997–2000	308 (296–320)	725 (669–780)	246 (235–258)	2.95 (2.69–3.22)
2001–2004	303 (291–314)	629 (584–675)	243 (231–254)	2.59 (2.38–2.83)
2005–2008	267 (256–277)	578 (532–624)	216 (206–227)	2.67 (2.43–2.93)
2009–2012	258 (248–268)	581 (534–628)	210 (200–220)	2.76 (2.51–3.04)
2013–2016	236 (226–245)	436 (399–473)	204 (194–214)	2.14 (1.94–2.36)
**Men - stratified by period**				
1985–1988	543 (517–570)	725 (644–807)	511 (483–539)	1.42 (1.25–1.61)
1989–1992	515 (490–540)	797 (717–877)	464 (438–490)	1.72 (1.53–1.93)
1993–1996	440 (419–462)	770 (697–844)	381 (359–403)	2.02 (1.81–2.26)
1997–2000	456 (435–478)	966 (881 − 1.050)	375 (355–396)	2.57 (2.32–2.85)
2001–2004	453 (433–473)	860 (790–930)	372 (352–393)	2.31 (2.09–2.55)
2005–2008	398 (379–416)	770 (701–839)	330 (312–349)	2.33 (2.10–2.59)
2009–2012	384 (367–402)	802 (730–874)	313 (295–331)	2.56 (2.30–2.85)
2013–2016	355 (338–372)	602 (542–661)	313 (296–331)	1.92 (1.71–2.15)
**Women - stratified by period**				
1985–1988	185 (172–199)	496 (418–575)	137 (125–150)	3.61 (3.00-4.33)
1989–1992	205 (191–219)	511 (438–583)	159 (146–173)	3.21 (2.72–3.79)
1993–1996	180 (167–192)	495 (424–566)	137 (125–149)	3.61 (3.05–4.27)
1997–2000	168 (155–180)	497 (424–569)	124 (113–135)	4.01 (3.38–4.76)
2001–2004	160 (149–172)	410 (352–469)	119 (108–131)	3.44 (2.90–4.07)
2005–2008	143 (132–154)	395 (334–457)	108 (98–118)	3.66 (3.05–4.39)
2009–2012	139 (128–149)	371 (310–433)	113 (103–123)	3.29 (2.72–3.97)
2013–2016	123 (113–133)	278 (233–324)	100 (90–109)	2.79 (2.31–3.37)
**First non-fatal MI**				
	**IRt**	**IRd**	**IRn**	**IRR**
**All years combined**				
Total	194 (191–197)	363 (350–377)	167 (163–170)	2.18 (2.09–2.28)
Men	301 (295–307)	488 (469–507)	265 (259–271)	1.84 (1.76–1.93)
Women	94 (91–97)	249 (231–268)	75 (72–78)	3.33 (3.06–3.63)
**Men and women - stratified by period**				
1985–1988	206 (194–217)	309 (269–349)	189 (177–200)	1.64 (1.42–1.89)
1989–1992	192 (181–202)	297 (260–334)	176 (166–187	1.68 (1.46–1.94)
1993–1996	171 (162–180)	304 (268–341)	152 (143–162)	2.00 (1.75–2.29)
1997–2000	174 (165–183)	398 (355–441)	143 (134–152)	2.78 (2.46–3.15)
2001–2004	218 (208–227)	443 (404–483)	179 (169–188)	2.48 (2.23–2.75)
2005–2008	206 (197–215)	441 (400–483)	170 (161–179)	2.60 (2.33–2.89)
2009–2012	198 (189–207)	441 (400–483)	163 (154–172)	2.71 (2.43–3.02)
2013–2016	189 (181–198)	333 (300–366)	168 (159–177)	1.99 (1.78–2.22)
**Men - stratified by period**				
1985–1988	329 (309–350)	397 (337–457)	317 (295–339)	1.25 (1.06–1.48)
1989–1992	287 (269–306)	373 (319–428)	273 (253–292)	1.37 (1.16–1.61
1993–1996	257 (241–273)	362 (312–412)	238 (221–255)	1.52 (1.30–1.78)
1997–2000	275 (259–291)	574 (508–640)	229 (213–245)	2.51 (2.19–2.87)
2001–2004	328 (311–346)	599 (540–659)	277 (259–294)	2.17 (1.93–2.44)
2005–2008	311 (294–327)	578 (517–639)	265 (248–281)	2.18 (1.93–2.47)
2009–2012	300 (284–316)	628 (563–693)	247 (231–262)	2.55 (2.26–2.88)
2013–2016	291 (275–306)	473 (420–526)	262 (246–278)	1.81 (1.59–2.05)
**Women - stratified by period**				
1985–1988	88 (79–98)	226 (173–278)	67 (58–76)	3.38 (2.58–4.42)
1989–1992	101 (91–111)	225 (173–276)	85 (75–95)	2.63 (2.04–3.41)
1993–1996	90 (81–99)	250 (197–303)	71 (62–79)	3.53 (2.76–4.51)
1997–2000	78 (70–86)	232 (176–288)	62 (54–70)	3.75 (2.85–4.92)
2001–2004	113 (103–122)	295 (242–348)	86 (76–95)	3.44 (2.79–4.25)
2005–2008	107 (97–116)	312 (256–368)	80 (71–89)	3.88 (3.14–4.79)
2009–2012	101 (92–109)	264 (211–317)	83 (74–92)	3.17 (2.53–3.98)
2013–2016	93 (85–102)	200 (161–239)	78 (70–87)	2.57 (2.05–3.21)
**Fatal MI**				
	**IRt**	**IRd**	**IRn**	**IRR**
**All years combined**				
Total	99 (96–101)	209 (199–218)	78 (76–81)	2.67 (2.53–2.82)
Men	140 (136–144)	256 (242–269)	115 (111–119)	2.22 (2.09–2.37)
Women	62 (59–64)	167 (153–180)	45 (43–47)	3.70 (3.37–4.07)
**Men and women - stratified by period**				
1985–1988	154 (144–164)	299 (259–339)	131 (121–141)	2.28 (1.96–2.66)
1989–1992	164 (154–173)	353 (314–392)	131 (121–140)	2.70 (2.36–3.08)
1993–1996	135 (127–144)	325 (289–360)	104 (96–111)	3.14 (2.75–3.58)
1997–2000	134 (126–142)	327 (292–361)	103 (95–110)	3.17 (2.79–3.61)
2001–2004	85 (79–91)	186 (164–208)	64 (58–70)	2.91 (2.50–3.38)
2005–2008	61 (56–66)	136 (116–157)	46 (41–51)	2.95 (2.46–3.54)
2009–2012	61 (56–66)	140 (117–162)	48 (43–52)	2.93 (2.43–3.55)
2013–2016	46 (42–51)	103 (85–120)	36 (32–40)	2.83 (2.31–3.48)
**Men - stratified by period**				
1985–1988	214 (197–231)	329 (273–384)	194 (177–212)	1.69 (1.40–2.05)
1989–1992	227 (210–244)	424 (364–483)	191 (174–208)	2.22 (1.88–2.62)
1993–1996	184 (170–198)	409 (355–462)	143 (130–156)	2.86 (2.43–3.36)
1997–2000	181 (168–195)	392 (339–444)	146 (133–159)	2.68 (2.29–3.15)
2001–2004	125 (114–135)	260 (223–298)	96 (85–106)	2.72 (2.28–3.26)
2005–2008	87 (78–95)	192 (160–225)	66 (58–74)	2.93 (2.37–3.61)
2009–2012	84 (76–93)	174 (142–205)	66 (58–75)	2.62 (2.11–3.26)
2013–2016	64 (57–71)	129 (103–155)	51 (44–59)	2.50 (1.95–3.20)
**Women - stratified by period**				
1985–1988	97 (87–107)	270 (212–329)	71 (62–80)	3.83 (2.98–4.92)
1989–1992	104 (94–114)	286 (235–338)	74 (65–83)	3.87 (3.11–4.82)
1993–1996	90 (81–99)	245 (198–292)	66 (58–74)	3.71 (2.95–4.66)
1997–2000	90 (81–99)	265 (219–311)	62 (54–70)	4.27 (3.44–5.32)
2001–2004	48 (41–54)	115 (90–140)	34 (28–40)	3.41 (2.58–4.51)
2005–2008	36 (31–41)	83 (58–109)	28 (23–33)	3.01 (2.10–4.31
2009–2012	38 (33–44)	107 (76–139)	30 (25–35)	3.60 (2.55–5.07)
2013–2016	29 (25–34)	78 (55–101)	22 (17–26)	3.58 (2.49–5.16)

^a^ Incidence Rates, standardised to the German population 2000

IRt, all cases of first MI in total population; IRd, cases of first MI in individuals with diabetes in population with diabetes; IRn, cases of first MI in individuals without diabetes in population without diabetes; IRR, incidence rate ratio (IRd/IRn)

### Time trend analysis

#### First MI

In the male population with diabetes, the IR in 1997–2000 was significantly higher than in the initial study period and only decreased significantly in the last period (see Table 3). In the female population with diabetes, a significant decrease of IR to about the half of the initial value in 1985–1988 was observed in the last three periods. In the male population without diabetes a consistent decline of 25% was found from the mid-90s and continued until the last period. A similar decline only occurred in the female population in the last three periods.

**Table 3:** Results of Poisson models for people with and without diabetes, Augsburg, MONICA/KORA study region, 1985–2016

First MI (both non-fatal MI and fatal MI)			
	Relative risk for first MI (95% CI)^d^		
	Both sexes	Men	Women
Model 1a (population with diabetes)			
Time period^a^			
1989–1992	0.99 (0.82–1.20)	1.03 (0.76–1.39)	0.97 (0.79–1.19)
1993–1996	1.00 (0.82–1.20)	1.06 (0.79–1.44)	0.91 (0.74–1.13)
1997–2000	1.19 (1.002–1.44)*	1.35 (1.01–1.81)*	0.99 (0.80–1.22)
2001–2004	1.04 (0.87–1.25)	1.20 (0.91–1.60)	0.84 (0.68–1.04)
2005–2008	0.85 (0.71–1.03)	1.00 (0.75–1.33)	0.66 (0.53–0.82)*
2009–2012	0.81 (0.67–0.97)*	0.95 (0.72–1.26)	0.60 (0.48–0.75)*
2013–2016	0.63 (0.52–0.77)*	0.72 (0.54–0.97)*	0.52 (0.41–0.65)*
Male vs. female	1.88 (1.71–2.07)*		
Age (years) ^b^			
65–74	3.45 (2.98–3.99)*	3.54 (2.90–4.31)*	2.59 (2.08–3.22)*
55–64	1.79 (1.53–2.08)*	1.92 (1.56–2.36)*	1.23 (0.97–1.56)
Model 1b (population without diabetes)			
Time period^a^			
1989–1992	0.97 (0.87–1.09)	0.91 (0.83-0.9997)*	1.16 (0.99–1.35)
1993–1996	0.81 (0.73–0.91)*	0.75 (0.68–0.83)*	1.00 (0.85–1.17)
1997–2000	0.78 (0.70–0.87)*	0.74 (0.68–0.81)*	0.90 (0.77–1.06)
2001–2004	0.77 (0.69–0.86)*	0.74 (0.67–0.81)*	0.87 (0.74–1.03)
2005–2008	0.69 (0.61–0.77)*	0.65 (0.60–0.72)*	0.80 (0.67–0.94)*
2009–2012	0.67 (0.60–0.75)*	0.62 (0.57–0.69)*	0.83 (0.71–0.98)*
2013–2016	0.65 (0.58–0.73)*	0.62 (0.57–0.69)*	0.74 (0.62–0.87)*
Male vs. female	3.24 (3.04–3.45)*		
Age (years) ^b^			
65–74	4.07 (3.78–4.38)*	3.41 (3.20–3.62)*	6.97 (6.17–7.89)*
55–64	2.06 (1.91–2.23)*	1.97 (1.85–2.10)*	2.53 (2.21–2.90)*
Models - stratified for time period^c^			
Diabetes (yes vs. no)			
1985–1988	1.95 (1.62–2.34)*	1.40 (1.22–1.61)*	3.33 (2.84–3.91)*
1989–1992	2.01 (1.70–2.37)*	1.61 (1.33–1.96)*	2.87 (2.34–3.52)*
1993–1996	2.37 (2.04–2.75)*	2.00 (1.72–2.33)*	3.14 (2.49–3.95)*
1997–2000	2.93 (2.55–3.38)*	2.60 (2.26–2.99)*	3.71 (3.19–4.33)*
2001–2004	2.61 (2.24–3.03)*	2.33 (2.03–2.67)*	3.35 (2.61–4.29)*
2005–2008	2.46 (2.13–2.84)*	2.24 (1.91–2.63)*	3.07 (2.42–3.91)*
2009–2012	2.42 (2.09–2.80)*	2.33 (2.00-2.71)*	2.72 (2.08–3.56)*
2013–2016	1.92 (1.63–2.27)*	1.75 (1.47–2.09)*	2.57 (2.03–3.25)*
**First non-fatal M**I			
	**Relative risk for first non-fatal MI (95% CI)**^**d**^		
	**Both sexes**	**Men**	**Women**
Model 1a (population with diabetes)			
Time period^a^			
1989–1992	0.86 (0.68–1.09)	0.87 (0.62–1.22)	0.86 (0.63–1.17)
1993–1996	0.92 (0.73–1.15)	0.92 (0.66–1.27)	0.92 (0.68–1.24)
1997–2000	1.23 (0.99–1.53)	1.46 (1.08–1.98)*	0.85 (0.62–1.17)
2001–2004	1.41 (1.15–1.73)*	1.54 (1.15–2.05)*	1.22 (0.92–1.62)
2005–2008	1.28 (1.04–1.57)*	1.41 (1.05–1.88)*	1.09 (0.82–1.43)
2009–2012	1.23 (1.01–1.51)*	1.42 (1.07–1.88)*	0.92 (0.69–1.23)
2013–2016	0.98 (0.79–1.20)	1.09 (0.81–1.46)	0.79 (0.59–1.07)
Male vs. female	2.02 (1.82–2.24)*		
Age (years)^b^			
65–74	2.26 (1.96–2.61)*	2.29 (1.91–2.75)*	1.83 (1.41–2.36)*
55–64	1.49 (1.28–1.73)*	1.57 (1.30–1.89)*	1.11 (0.84–1.46)
Model 1b (population without diabetes)			
Time period^a^			
1989–1992	0.96 (0.84–1.09)	0.87 (0.78–0.97)*	1.28 (1.03–1.58)*
1993–1996	0.82 (0.72–0.93)*	0.76 (0.68–0.85)*	1.07 (0.86–1.33)
1997–2000	0.77 (0.68–0.88)*	0.73 (0.65–0.82)*	0.94 (0.75–1.17)
2001–2004	0.96 (0.85–1.08)	0.88 (0.79–0.98)*	1.29 (1.05–1.59)*
2005–2008	0.91 (0.81–1.03)	0.84 (0.76–0.94)*	1.22 (0.99–1.51)
2009–2012	0.88 (0.78–0.99)*	0.79 (0.71–0.88)*	1.26 (1.03–1.55)*
2013–2016	0.90 (0.80–1.02)	0.83 (0.75–0.92)*	1.19 (0.96–1.46)
Male vs. female	3.59 (3.34–3.85)*		
Age (years) ^b^			
65–74	2.99 (2.77–3.23)*	2.53 (2.37–2.71)*	5.10 (4.44–5.86)*
55–64	1.90 (1.75–2.06)*	1.82 (1.70–1.95)*	2.32 (1.99–2.69)*
Models - stratified for time period^c^			
Diabetes (yes vs. no)			
1985–1988	1.70 (1.37–2.11)*	1.24 (1.05–1.46)*	3.22 (2.53–4.10)*
1989–1992	1.56 (1.27–1.92)*	1.27 (0.98–1.64)	2.29 (1.78–2.95)*
1993–1996	1.89 (1.56–2.29)*	1.51 (1.30–1.75)*	2.93 (2.12–4.06)*
1997–2000	2.69 (2.24–3.23)*	2.51 (2.00-3.16)*	3.22 (2.39–4.33)*
2001–2004	2.44 (2.08–2.87)*	2.17 (1.90–2.47)*	3.24 (2.40–4.36)*
2005–2008	2.36 (2.00-2.79)*	2.09 (1.76–2.49)*	3.21 (2.40–4.29)*
2009–2012	2.38 (2.03–2.80)*	2.31 (1.95–2.74)*	2.63 (1.95–3.56)*
2013–2016	1.79 (1.52–2.11)*	1.65 (1.38–1.97)*	2.38 (1.87–3.03)*
**Fatal MI**			
	**Relative risk for fatal MI (95% CI)**^**d**^		
	**Both sexes**	**Men**	**Women**
Model 1a (population with diabetes)			
Time period^a^			
1989–1992	1.15 (0.95–1.40)	1.25 (0.93–1.69)	1.06 (0.83–1.35)
1993–1996	1.08 (0.89–1.31)	1.24 (0.93–1.67)	0.91 (0.71–1.17)
1997–2000	1.15 (0.95–1.40)	1.21 (0.90–1.63)	1.11 (0.87–1.42)
2001–2004	0.68 (0.55–0.83)*	0.81 (0.60–1.09)	0.52 (0.39–0.69)*
2005–2008	0.44 (0.35–0.55)*	0.55 (0.40–0.75)*	0.31 (0.23–0.43)*
2009–2012	0.40 (0.32–0.50)*	0.45 (0.33–0.62)*	0.34 (0.25–0.47)*
2013–2016	0.30 (0.24–0.38)*	0.32 (0.23–0.46)*	0.28 (0.20–0.40)*
Male vs. female	1.75 (1.57–1.95)*		
Age (years) ^b^			
65–74	7.83 (6.31–9.72)*	8.78 (6.68–11.53)*	4.60 (3.20–6.62)*
55–64	2.78 (2.21–3.49)*	3.16 (2.37–4.22)*	1.57 (1.06–2.33)*
Model 1b (population without diabetes)			
Time period^a^			
1989–1992	1.00 (0.88–1.13)	0.98 (0.85–1.12)	1.04 (0.86–1.26)
1993–1996	0.80 (0.70–0.90)*	0.74 (0.64–0.85)*	0.93 (0.77–1.14)
1997–2000	0.79 (0.70–0.89)*	0.76 (0.66–0.87)*	0.87 (0.71–1.06)
2001–2004	0.49 (0.43–0.56)*	0.49 (0.43–0.58)*	0.47 (0.37–0.60)*
2005–2008	0.35 (0.31–0.41)*	0.34 (0.29–0.40)*	0.39 (0.31–0.50)*
2009–2012	0.37 (0.32–0.43)*	0.35 (0.29–0.41)*	0.42 (0.34–0.54)*
2013–2016	0.28 (0.24–0.33)*	0.27 (0.23–0.32)*	0.31 (0.24–0.40)*
Male vs. female	2.66 (2.47–2.87)*		
Age (years) ^b^			
65–74	8.60 (7.70–9.59)*	7.30 (6.51–8.18)*	13.28 (10.74–16.41)*
55–64	2.77 (2.46–3.13)*	2.65 (2.35-3.00)*	3.28 (2.59–4.14)*
Models - stratified for time period^c^			
Diabetes (yes vs. no)			
1985–1988	2.28 (1.90–2.73)*	1.68 (1.35–2.08)*	3.43 (2.76–4.27)*
1989–1992	2.61 (2.22–3.06)*	2.15 (1.86–2.49)*	3.48 (2.62–4.62)*
1993–1996	3.03 (2.55–3.59)*	2.84 (2.26–3.57)*	3.35 (2.53–4.43)*
1997–2000	3.23 (2.73–3.83)*	2.72 (2.24–3.31)*	4.15 (3.46–4.98)*
2001–2004	3.04 (2.50–3.70)*	2.79 (2.20–3.53)*	3.60 (2.66–4.86)*
2005–2008	2.76 (2.30–3.32)*	2.76 (2.14–3.55)*	2.73 (2.07–3.60)*
2009–2012	2.53 (2.08–3.08)*	2.38 (1.98–2.86)*	2.93 (1.94–4.41)*
2013–2016	2.51 (1.95–3.23)*	2.26 (1.69–3.02)*	3.20 (2.16–4.74)*

*p < 0.05

^a^ Reference: 1985–1988

^b^ Reference: 45–54 years

^c^ Adjusted for age and sex

^d^ 95% Confidence interval

The RR comparing men with and without diabetes significantly increased from 1.40 (1.22–1.61) in the years 1985–1988 to 2.60 (2.26–2.99) in 1997–2000 (p-value comparing the RR with those in 1985–1988 from the interaction model < 0.001) and decreased thereafter to 1.75 (1.47–2.09) in 2013–2016 (*p* = 0.27). In women the RR significantly decreased only during the last study period (*p* = 0.04).

#### First non-fatal MI

In the male population with diabetes a significant increase of first non-fatal MI IR was observed in the late 1990s, with a slight decrease during the time period 2013–2016 (see Table 3). In men without diabetes, IR decreased in the early 1990s and subsequently plateaued until 2013–2016. In women with and without diabetes no consistent time trend was identified.

The time trend for RR (diabetes vs. no diabetes) in men with first non-fatal MI was quite similar to that in men with first MI (both non-fatal and fatal). No significant decrease of RR was observed throughout the entire study period in women with first non-fatal MI.

#### Fatal MI

A marked decrease of fatal MI IR of about 70% of the initial value in 1985–1988 was observed in the population with diabetes in both sexes beginning in the 2000s (see Table 3). Likewise, in the population without diabetes a consistent and similar decline of IR was seen in both sexes, starting in the 1990s in men.

In both sexes, the RR (diabetes vs. no diabetes) for fatal MI was somewhat higher than for first non-fatal MI. The RR again significantly increased among men from 1.68 (1.35–2.08) in 1985–1988 to 2.84 (2.26–3.57) in 1993–1996 (*p* < 0.001) and decreased thereafter, while no consistent change of RR was seen in women.

### Age and sex

The risk of first non-fatal MI and fatal MI was significantly associated with male sex and increasing age in all models, the latter association being particularly strong regarding fatal MI (see Table 3).

### Sensitivity analyses

The results of the sensitivity analysis revealed similar time trends when diabetes prevalence was estimated solely from KORA surveys. Moreover, in the population with diabetes the significant decline of IR of first MI, first non-fatal MI, and fatal MI was confirmed in the 2009–2016 sensitivity analysis as only there could diabetes prevalence be correctly estimated for each year.

## Discussion

### Main findings

IR of first MI in the Augsburg region remained significantly higher in people with diabetes than without between 1985 and 1988 and 2013–2016, being true for both sexes regardless of fatality of first MI. Compared to the first study period, a significant decrease in IR of first MI was observed in women and men with and without diabetes. However, the decrease of IR of first MI was delayed in men with diabetes, due to an increase of first non-fatal MI until the late 2000s, followed by a slight decrease only in the last study period (2013–2016). The RR comparing men with and without diabetes substantially increased until the year 2000 and decreased thereafter, both for fatal and first non-fatal MI. However, no consistent time trends were seen among women regardless of first MI fatality.

### Comparison with other studies

#### First MI

Our previous study reported a significant decrease of IR among women with diabetes and men and women without diabetes, but a significant increase in men with diabetes between 1985 and 2006 [11].

The current study found a significant decrease in all subgroups, including in men with diabetes since the 2010ies. Primary and secondary MI prevention, evidence-based implementation of more widespread and intensified use of antihypertensive medication, lipid lowering therapy and platelet inhibition as well as better treatment of coronary heart disease such as percutaneous coronary intervention might explain these decreasing incidences [25, 26]. Only a few population-based studies have reported time trends for first MI including both first non-fatal MI and fatal MI in people with and without diabetes jointly with contradictory results. Our findings are well in line with Swedish study findings showing a clear decrease of IR only in men without diabetes and plateaued IR in men with diabetes and women with and without diabetes in the 1990s [9]. A Finnish [7] and a U.S. study [8] both reported differences between sexes and found a decline of IR among men with diabetes and among men and women without diabetes, but no change in women with diabetes. Unlike our findings, a recent Scottish study reported simultaneous reduction of IR per calendar year in people with and without diabetes between 2005 and 2016 without sex differences with unchanged RR during the study period [10]. Like our study, a more recent Swedish study reported a decreasing RR between the populations with and without diabetes between 1998 and 2012 [27]. However, our study covering 1985–2016 found no significant reduction of RR of first MI between people with and without diabetes. The overarching St. Vincent goal of RR reduction was therefore not achieved in the study region during the 30-year study period.

#### First non-fatal MI

No material changes in IR of first non-fatal MI were found in women with diabetes or in men and women without diabetes. However, in men with diabetes, IRs of first non-fatal MI increased until 2009–2012, followed by a slight decrease in 2013–2016. A study from England reported unchanged hospitalization rates for MI in people with diabetes but declined rates in people without diabetes in the period 2004–2014, with similar trends in men and women [5]. In contrast, a recent study from Sweden found significantly decreased incidence rates in people with type 2 diabetes and matched controls without diabetes during the period 2001–2019 [28]. A U.S. study reported considerable declines in hospitalization rates of MI among people with diabetes, less pronounced reductions in people without diabetes, and reduced RRs between 1990 and 2010 [29]. Interestingly, a subsequent U.S. study reported plateaued hospitalization rates of MI in people with diabetes aged 65 + but increased rates in young people between 2010 and 2015 [4]. An Australian study found significantly reduced MI hospitalization rates in people with diabetes between 1998 and 2010, but not in people without diabetes, resulting in a corresponding reduction of RR [6]. However, none of these studies are fully comparable to our study due to study design differences.

#### Fatal MI

The proportion of fatal MI dropped substantially in people with and without diabetes. However, when taking all people (with and without diabetes) with fatal MI into account, the percentage of people with diabetes during the study period increased from 28% at the start to 37% at the end of the study period.

We observed a decrease of IR of fatal MI in both men and women regardless of diabetes status. The significant reduction of IR of fatal MI since the early 2000s may be explained by improved MI treatment methods, such as routine use of primary percutaneous coronary intervention (with stents) in people with MI resulting in better survival [25, 26, 30]. To the best of our knowledge, very few studies have analyzed time trends for fatal MI in people with and without diabetes with a comparable study design. Two Finnish studies analyzed CHD mortality time trends among people with and without diabetes [7, 31]. The earlier study analyzed fatal CHD until 1997, reporting decreased CHD mortality in people with and without diabetes. RRs comparing mortality rates between these populations remained unchanged in men and were substantially reduced in women for a 10-year follow-up period [7]. However, a more recent study reported declined mortality rates between 1997 and 2010 in people with and without diabetes, and a reduced secular trend of RRs between these populations [31]. A Swedish study found a significant reduction of about 50% of fatal CHD incidence in people with type 2 diabetes as well as in matched controls between 1998 and 2014. However, this decline was less pronounced in people with type 2 diabetes than in controls [32]. Nationwide findings from the U.S. demonstrated a substantial decrease in mortality due to heart diseases both in people with and without diabetes between 1988 and 2015 [33] In all of these studies, CHD mortality was about twice as high in people with diabetes than those without, which is well in line with our results [7, 31–33].

### Sex disparities

Irrespective of diabetes status and type of first MI, men have a higher absolute risk for first non-fatal MI and fatal MI. However, the impact of diabetes was consistently greater among women. The RR comparing people with and without diabetes regarding first MI was 3.5 in women and 2.0 in men. Our results are in line with other studies’ findings [10, 27, 34]. A recent French paper showed that the RR of cardiovascular complications associated with diabetes was generally higher in women than in men, with the strongest effect in patients with MI [35]. Several factors might explain these unfavorable sex disparities, such as women with diagnosed diabetes having a worse metabolic and cardiovascular profile than those without [36]. Some studies already identified these unfavorable changes in women with pre-diabetes [37].

### Limitations and strengths

One study limitation is the findings’ dependency on correct estimations of the study region’s diabetic population at risk. We therefore combined two data sources. One used self-reported data from the study region, which partly underestimated diabetes prevalence [38]; the other used regional administrative data (Zi-data) excluding privately insured people and people without a medical consultation. For the purpose of estimating diabetes prevalence in the study region we connected these data sources using correction factors, which might lead to an over or underestimation of diabetes prevalence, especially in the early study period. However, we believe that this approach is valid since Zi-data covers more than 80% of the regional population and changes in diabetes prevalence in the study region in the group aged 45 to 74 between 1985 and 2016 were only marginal. A second limitation arose from changed definitions of non-fatal MI during the study period, particularly the use of troponin markers for MI diagnosis since 2000. This might have led to an increase of less severe or atypical MI cases. However, the new definition also required a number of clinical symptoms and ECG diagnostic criteria, meaning no MIs were defined solely by troponin. Furthermore, the IR of first non-fatal MI in our study did not significantly increase in the early 2000s irrespective of diabetes status and sex, suggesting no substantial impact of changed diagnosis criteria on time trends.

Thirdly, the diagnostic criteria of diabetes were changed between 1997 and 1999 [39, 40] and again between 2009–2011 [41, 42]. Both changes were initiated by the American Diabetes Association and later adopted by the WHO. The revised definitions could increase the number of people diagnosed with diabetes at an earlier stage, and may thus lead to a lower incidence of MI. However, we believe that this change does not have a huge impact on our results since diabetes prevalence in the study population did not materially change between 1985 and 2016. Finally, we were only able to analyze the population aged 45–74 years despite significant MI rates in people aged over 75 [43]. This limitation was due to using KORA-surveys and the KORA myocardial infarction register, which primarily analyzes premature non-fatal MI and fatal MI in the population aged below 75. The regional nature of our study’s data, covering a population of approximately 400,000 people, posed a further limitation. Thus, our results can only be partially generalized to the whole German population.

The following strengths should, however, be emphasized. Foremost, to the best of our knowledge, except Saeed et al., [44] this study is the longest population-based study of non-fatal MI and fatal MI time trends (32 years). Secondly, our analysis used valid data from a well-organized population-based registry with an established algorithm for recording nearly all MIs in the study region. This approach is especially advantageous because it not only records MI hospitalizations but also CHD fatalities. Thirdly, we analyzed IR of first non-fatal MI and fatal MI in people with diabetes in relation to the population with diabetes at risk and thus considered the possible change of diabetes prevalence during the study period.

## Conclusion

The IR of first MI in people with diabetes remained substantially higher than in people without diabetes, which was also true for both first non-fatal MI and fatal MI. Compared to the first time period, the IR of first MI and fatal MI decreased significantly in the population with and without diabetes. The decrease of IR of first MI was delayed in men with diabetes due to an increase of first non-fatal MI until the late 2000s, followed by a slight decrease in the last study period. The RR comparing people with and without diabetes increased in men until the turn of the millennium and decreased thereafter, while no consistent change was seen among women irrespective of MI fatality. The decrease of first MI could be partly explained by treatment and management improvements. However, the gap between the populations with and without diabetes remained.

## Data Availability

The data and materials used in this study cannot be made available in the manuscript, the supplemental files, or in a public repository due to German data protection laws (*Bundesdatenschutzgesetz*). Therefore, they are stored on a secure drive in the German Diabetes Center to facilitate replication of the results. Generally, access to data of statutory health insurance funds for research purposes is possible only under the conditions defined in German Social Law (SGB V § 287). Requests for data access can be sent as a formal proposal specifying the recipient and purpose on the data transfer to the appropriate data protection agency. Access to the data used in this study can only be provided to external parties under the condition of the cooperation contract of this research project and after written approval by the KORA myocardial infarction registry (the data holder). For assistance in obtaining access to the data, please contact heiner.claessen@ddz.de.
